# Gastrostomy tract metastasis presenting as a large abdominal wall mass following percutaneous endoscopic gastrostomy for esophageal squamous cell carcinoma: a case report

**DOI:** 10.1093/jscr/rjaf510

**Published:** 2025-07-13

**Authors:** Jirat Leelapatanadit, Rawat Waratchanont, Wichitra Asanprakit, Viriya Kaewkangsadan, Sukchai Satthaporn

**Affiliations:** Department of Surgery, Phramongkutklao Hospital, Thung Phaya Thai, Ratchathewi, Bangkok, Thailand; Department of Surgery, Phramongkutklao Hospital, Thung Phaya Thai, Ratchathewi, Bangkok, Thailand; Department of Surgery, Phramongkutklao Hospital, Thung Phaya Thai, Ratchathewi, Bangkok, Thailand; Department of Surgery, Phramongkutklao Hospital, Thung Phaya Thai, Ratchathewi, Bangkok, Thailand; Department of Surgery, Phramongkutklao Hospital, Thung Phaya Thai, Ratchathewi, Bangkok, Thailand

**Keywords:** gastrostomy tract metastasis, gastrostomy tract seeding, PEG complication, PEG tract metastasis, PEG tract seeding, abdominal wall mass

## Abstract

Percutaneous endoscopic gastrostomy (PEG) is a standard method for providing enteral access in patients with obstructive aerodigestive cancer. However, gastrostomy tract metastasis is a rare but devastating complication in patient with aerodigestive cancers who have undergone PEG tube placement. Due to its rarity, the standard therapeutic approach remains undefined. We report the case of an 83-year-old male who developed gastrostomy tract metastasis following pull-type PEG tube placement, presenting as a large abdominal wall mass detected during surveillance following definite chemoradiation for locally advanced thoracic esophageal squamous cell carcinoma. The patient underwent en bloc resection of the abdominal wall mass along with the PEG tube. The abdominal wall defect was closed using an inter-layer polyglactin mesh repair, followed by delayed split-thickness skin grafting.

## Introduction

Percutaneous endoscopic gastrostomy (PEG) is a standard method for providing enteral access in patients with obstructive aerodigestive cancer and should be considered in those with swallowing difficulties who are expected to require nasogastric feeding tube for >4 weeks [1, 2]. Two methods are now used for PEG: the pull and push (or introducer) methods. Gastrostomy tract metastasis is a rare late complication after PEG placement, with an overall event rate of 0.4%–0.7% [3]. Due to its rarity, the standard therapeutic approach and prognosis remain undefined. We present a case of gastrostomy tract metastasis following pull-type PEG tube placement, which was treated with en bloc resection of the abdominal wall mass along with the PEG tube.

## Case report

**Figure 1:**
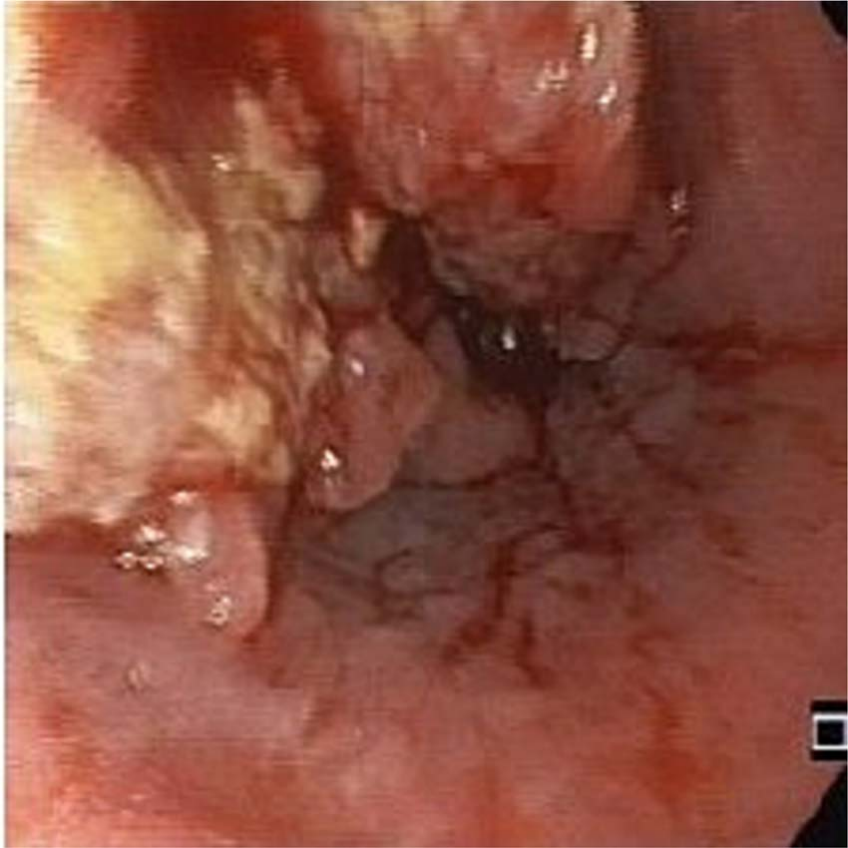
The primary esophageal tumor.

An 83-year-old male with progressive dysphagia and significant weight loss (15 kilograms in 3 months) was diagnosed with locally advanced upper thoracic esophageal squamous cell carcinoma, located 22–26 cm from the incisors, staged as T3N1M0 (Fig. 1). A PEG tube was inserted using the pull technique prior to initiating radiation (50.4 Gy) and chemotherapy (five cycles of Paclitaxel and Carboplatin). Three months after the initial diagnosis, he achieved a complete endoscopic and radiological response and regained the ability to eat orally.

**Figure 2:**
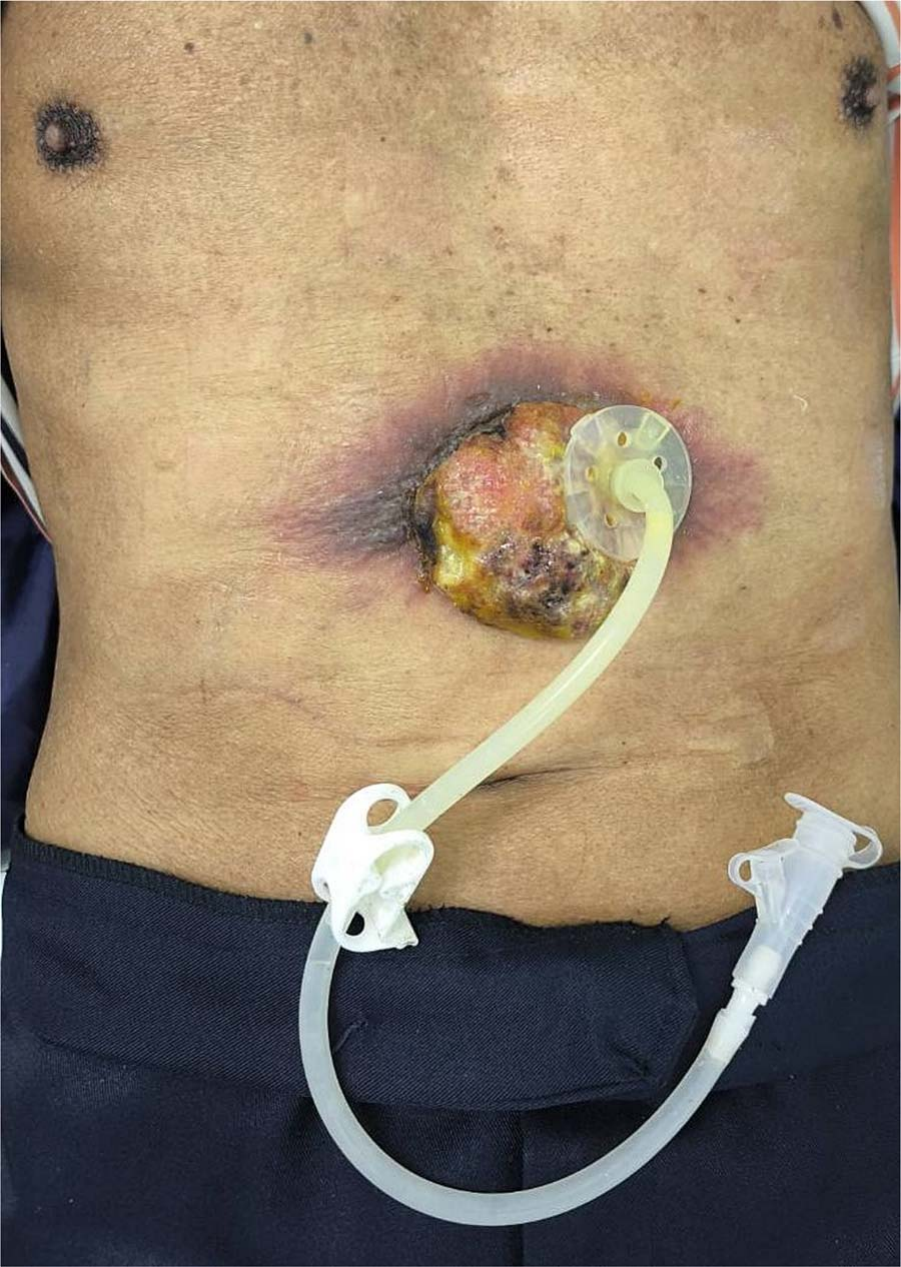
A large abdominal wall mass adjacent to the PEG tube site.

**Figure 3:**
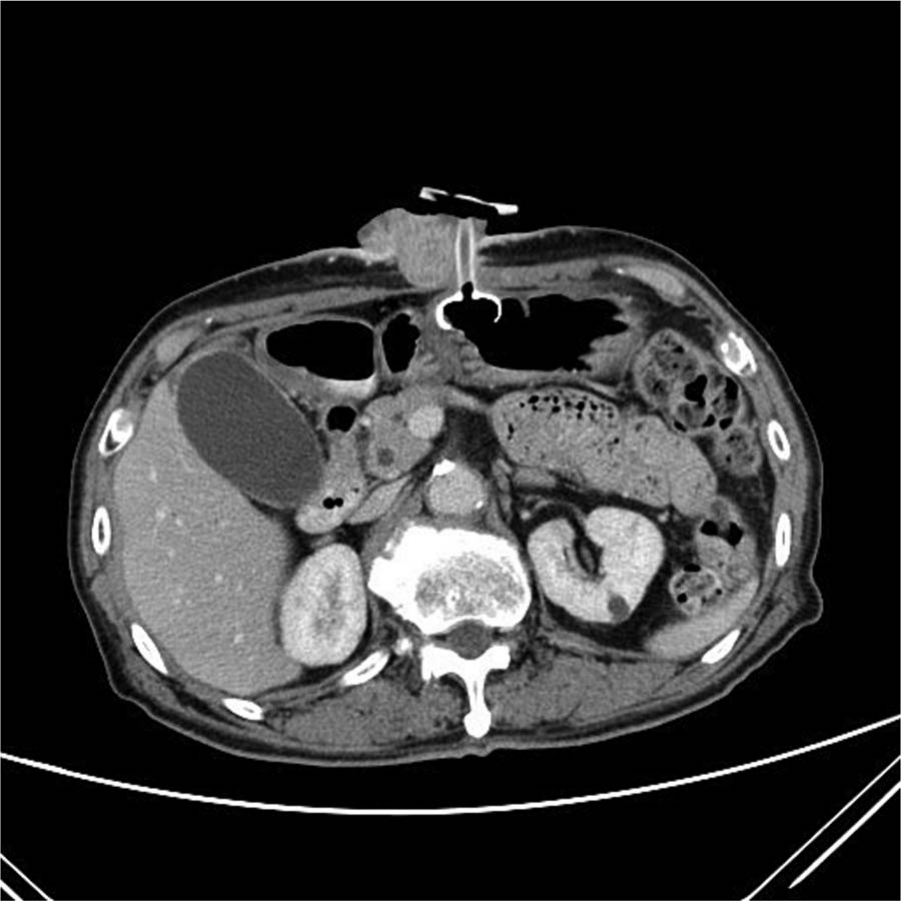
A CT scan of the abdomen showed a 6.2 x 5.6 cm enhancing soft tissue mass in the anterior abdominal wall.

Two months after completing chemoradiotherapy, a large abdominal wall mass measuring 6 x 6 cm with foul-smelling purulent discharge was noted adjacent to the PEG tube site (Fig. 2). Incisional biopsy of the mass was performed and revealed well-differentiated squamous cell carcinoma. According to these clinical and pathological findings, gastrostomy tract metastasis was suspected. A computed tomography (CT) scan of the chest and abdomen showed a 6.2 x 5.6 cm enhancing soft tissue mass in the anterior abdominal wall, centered around the gastrostomy site, extending deep to subcutaneous layer (Fig. 3). There was no evidence of distant metastasis. No mucosal lesion was observed on upper gastrointestinal endoscopy.

**Figure 4:**
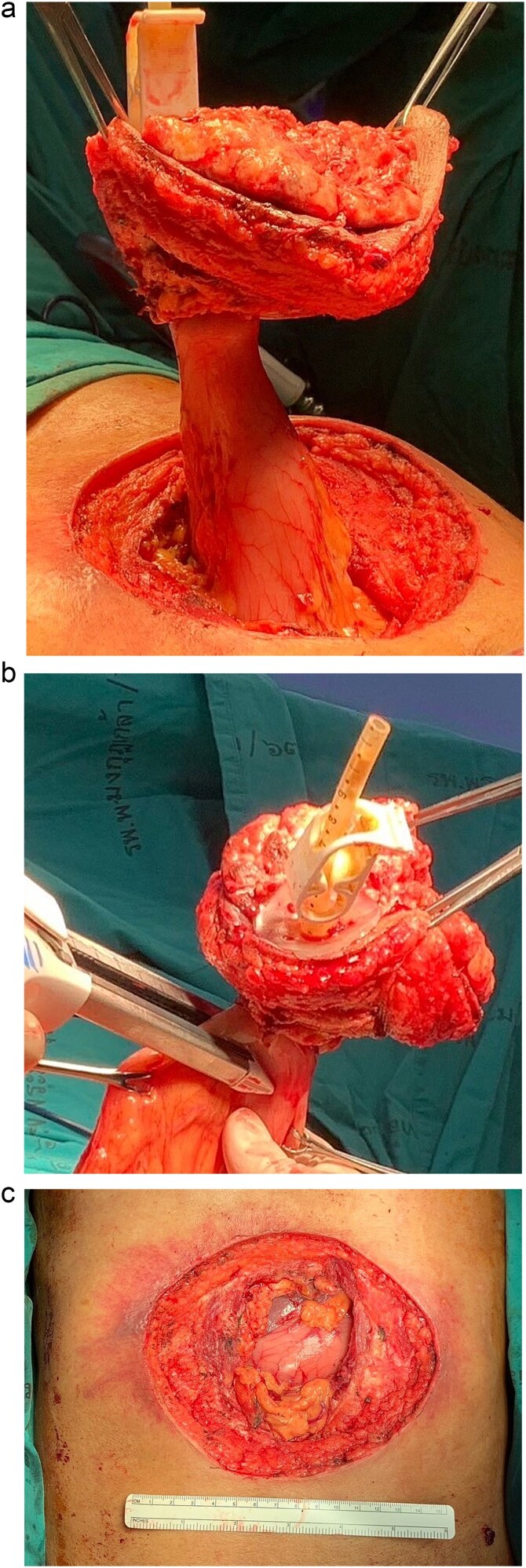
Operative procedure: (a) the mass and the PEG tube were dissected from the abdominal wall, (b) transected the stomach with surgical stapling devices, and (c) the abdominal wall defect.

**Figure 5:**
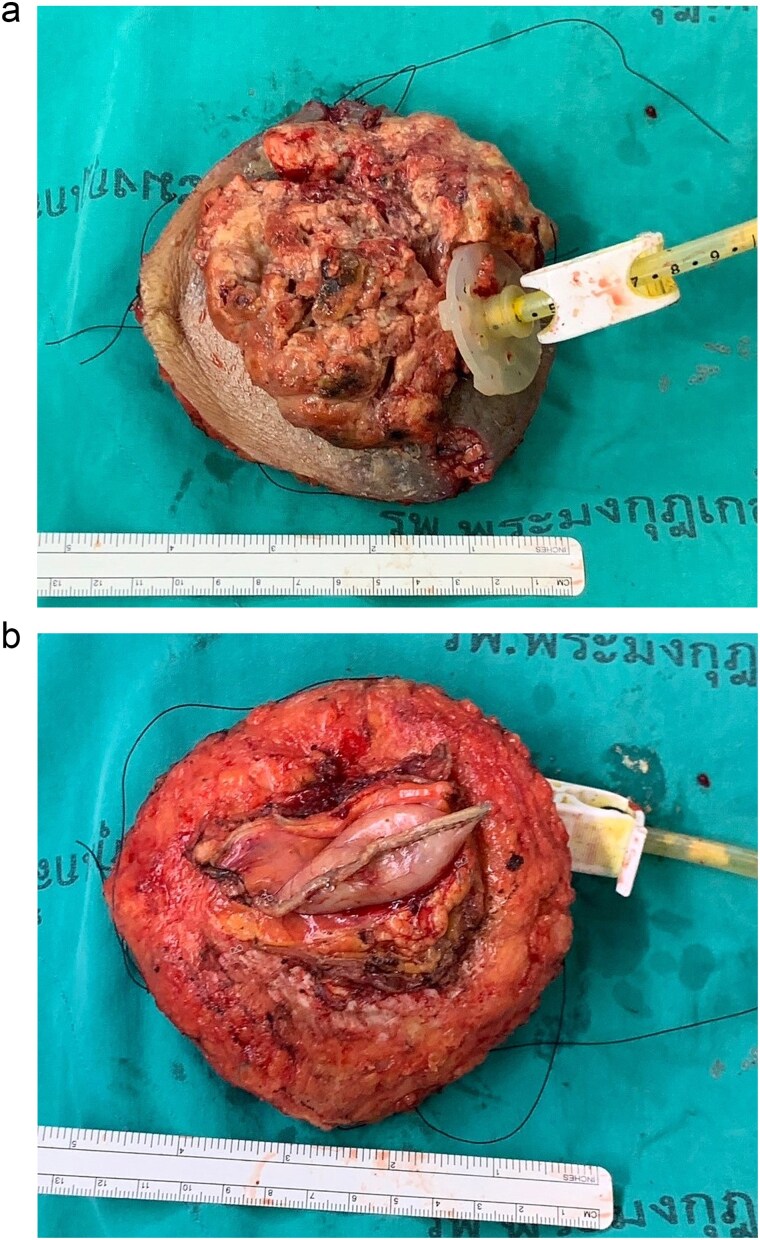
Gross specimen: (a) front, (b) back.

**Figure 6:**
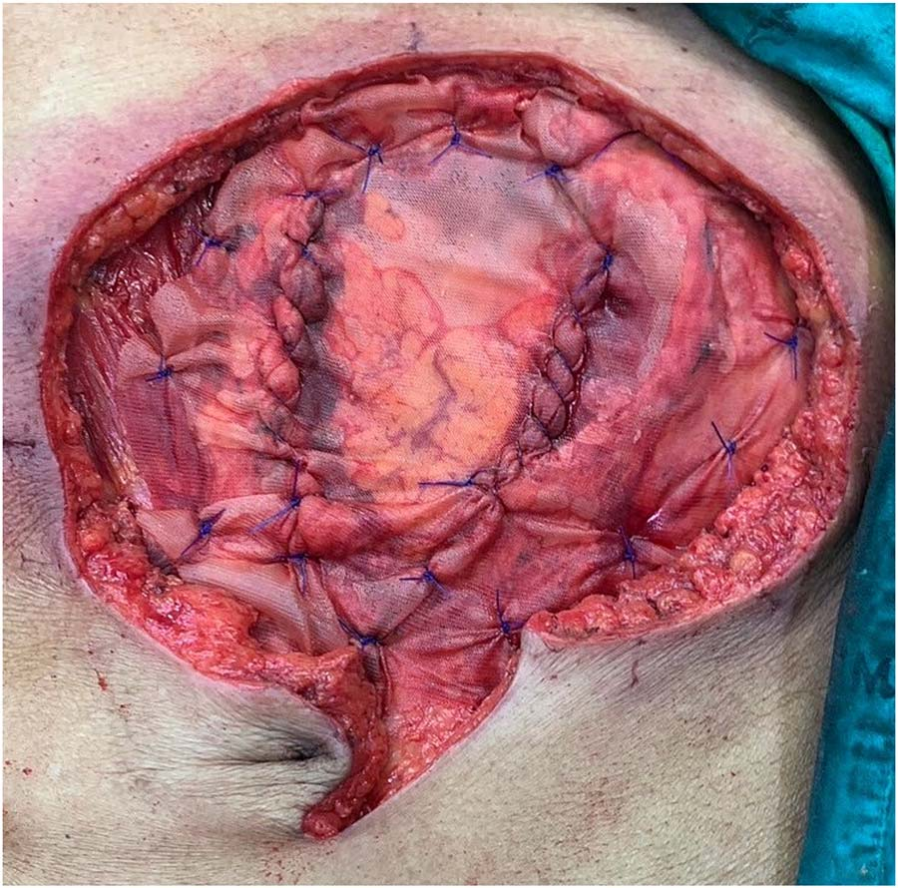
An inter-layer polyglactin mesh repair.

**Figure 7:**
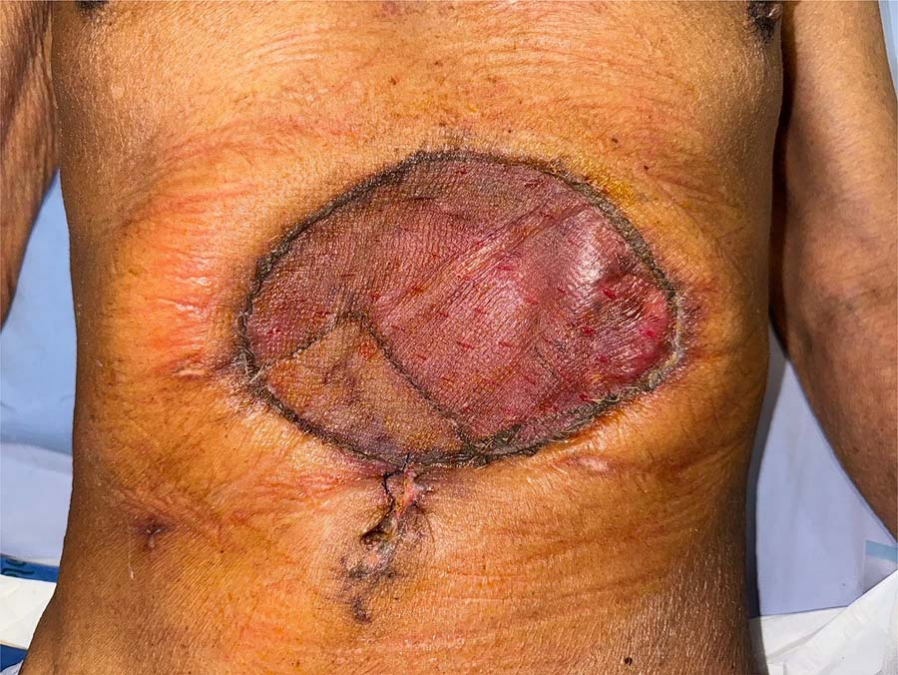
Two-week follow-up.

Following discussion of the treatment plan with the multidisciplinary team (oncologist, radiologist, pathologist, and plastic surgeon), the patient was scheduled to undergo en bloc resection of the mass with subsequent abdominal wall closure. The skin was incised around the mass with a gross margin of 2 cm. The abdominal wall was dissected deep to the rectus muscle, and the intra-abdominal cavity was entered around the mass and the PEG tract, and then transected the stomach with surgical stapling devices (Fig. 4). The specimen was sent to the pathologist for frozen section to evaluate the surgical margins (Fig. 5). After all surgical margins were confirmed negative by the pathologist, the abdominal wall defect was closed using an inter-layer polyglactin mesh repair (Fig. 6), and a vacuum dressing was applied. One week later, split-thickness skin grafting from the right thigh was performed at the well-granulated surgical wound bed. At the 2-week follow-up, the skin graft was fully taken (Fig. 7) and a long-term follow-up at 6 months showed no wound complication. The final pathological report revealed well-differentiated SCC and all margins were negative for malignancy.

## Discussion

Gastrostomy tract metastasis is a rare but serious complication following PEG placement, particularly in patients with upper aerodigestive tract and head and neck malignancies [3, 4]. The incidence ranges between 0.4% and 0.7%, with higher risk associated with advanced tumor stage and specific procedural techniques, especially the pull method [2, 3, 5]. In our case, the patient developed a large abdominal wall mass at the gastrostomy site two months after completion of chemoradiotherapy for esophageal SCC. Histopathological findings confirmed SCC at the PEG site, consistent with previous reports highlighting direct seeding as a likely mechanism of metastasis [3, 5].

The pull technique used in our patient has been associated with a higher risk of tumor seeding due to passage of the gastrostomy tube through the primary tumor site and potential for mucosal contamination [3, 5]. Siu *et al.* conducted a systematic review and meta-analysis and demonstrated that 97% of reported cases involved the pull technique, with squamous cell histology present in 95% of cases and most patients presenting with T3 or T4 disease [3]. Similarly, Butt and Reynolds reported a case of isolated PEG site metastasis from esophageal adenocarcinoma, supporting the theory of tumor implantation via the internal bolster of the PEG tube during insertion [6].

Due to the rarity of this condition, standardized treatment protocols remain undefined. Surgical en bloc resection is commonly employed for isolated metastasis [4, 6], as in our case, with the aim of achieving local control. However, long-term outcomes following resection remain unclear and merit further study. Future research should aim to evaluate oncologic outcomes such as disease-free and overall survival in patients undergoing surgery for PEG tract metastasis. Additionally, comparative studies assessing the role of multimodality treatment—including surgery, radiotherapy, and systemic therapy—are necessary to determine the most effective strategies for improving survival and quality of life in this subset of patients.
